# BCG immunotherapy for bladder cancer triggers systemic and local BCG-specific CD4^+^ Th1 responses

**DOI:** 10.1016/j.isci.2026.114676

**Published:** 2026-01-12

**Authors:** Paul Rollin, Benjamin Pluskwa, Emilie Artru, Tristan Le Vaslot, Daria Kartasheva-Ebertz, Diane Biron, Margaux Bossis, Fanny Onodi, Joel LeMaoult, Nathalie Rouas-Freiss, Mathieu F. Chevalier, Cecilia S. Lindestam Arlehamn, Alessandro Sette, Alexandra Masson-Lecomte, François Desgrandchamps, Evanguelos Xylinas, Pierre Tonnerre

**Affiliations:** 1 Institut de Recherche Saint-Louis, Université Paris-Cité, Inserm U1342, Paris, France; 2 Department of Urology, Bichat-Claude Bernard Hospital, Assistance-Publique Hôpitaux de Paris, Université Paris-Cité, Paris, France; 3 Department of Urology, Saint-Louis Hospital, Assistance-Publique Hôpitaux de Paris, Paris, France; 4 Hemato-Immunology Research Department, CEA, DRF-François Jacob Institute, Saint-Louis Hospital, Paris, France; 5 Center for Infectious Disease and Vaccine Research, La Jolla Institute for Immunology, La Jolla, San Diego, CA, USA; 6 Center for Vaccine Research, Department of Infectious Disease Immunology, Statens Serum Institut, Copenhagen, Denmark; 7 Department of Medicine, University of California, San Diego, San Diego, CA, USA

**Keywords:** immunology, oncology, therapeutics, immune response

## Abstract

High-risk non-muscle-invasive bladder cancer (NMIBC) is treated with intravesical instillations of Bacillus Calmette-Guérin (BCG), which trigger a local immune response. Improved patient outcomes have been linked to the recruitment of CD4^+^ T helper type 1 (Th1) cells to the bladder. However, specific antigens recognized by the bladder-infiltrating T cells remain largely unknown. In this study, we followed thirty-two patients with NMIBC undergoing BCG immunotherapy. Longitudinal blood and urine samples were collected to investigate the dynamics and characteristics of BCG-specific T cells. We detected pre-existing BCG-specific CD4^+^ T cells in most BCG therapy-naive patients before treatment induction. BCG immunotherapy increased the frequency and memory differentiation of circulating BCG-specific CD4^+^ T cells, which displayed a polyfunctional Th1 phenotype. Importantly, we provide evidence that BCG-specific CD4^+^ Th1 cells can be detected in urine during therapy, suggesting their recruitment to the bladder. This study provides novel biological insights into the cellular mechanisms of BCG-induced immunity in bladder cancer.

## Highlights

• Pre-existing BCG-specific CD4^+^ T cells are detectable in treatment-naive patients
• BCG therapy increases frequency and differentiation of BCG-specific CD4^+^ T cells
• BCG-specific CD4^+^ T cells show a polyfunctional Th1 cytokine profile
• BCG-specific CD4^+^ Th1 cells are detectable in urine during treatment

## Graphical abstract

**Figure:**
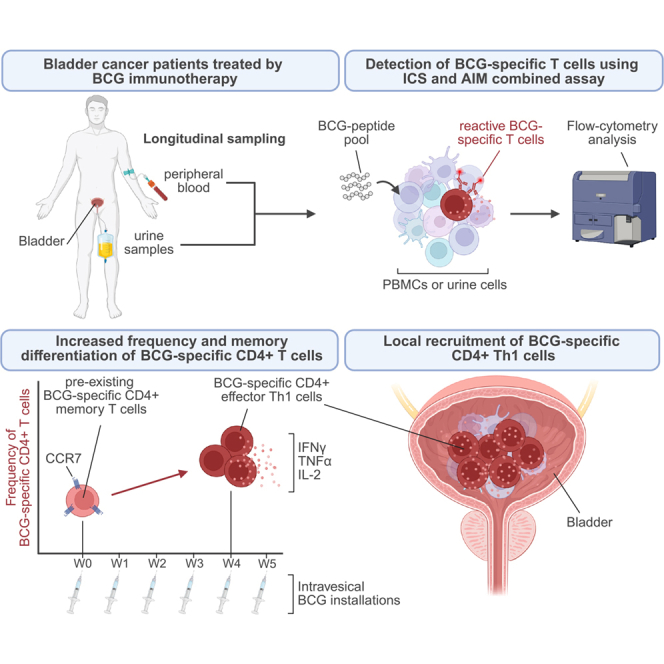

## Introduction

Bladder cancer is a prevalent and significant public health issue worldwide, ranking 9th among the most common cancers and 13th in terms of mortality.^1^ Anatomopathological analysis of resected tumors allows the disease to be classified into 2 groups—non-muscle-invasive bladder cancers (NMIBCs) and muscle-invasive bladder cancers (MIBCs)—with NMIBCs accounting for almost 80% of cases at diagnosis.^2^^,^^3^

High-risk NMIBCs are treated adjuvantly by endovesical instillations of Bacillus Calmette-Guérin (BCG).^3^^,^^4^^,^^5^ BCG immunotherapy consists of an induction phase of 6 instillations, followed by maintenance phases of 3 weekly instillations at 3 and 6 months, and then at every 6 months for up to 3 years. Previous studies have showed that BCG immunotherapy triggers a strong local immune response that intensifies with successive instillations, along with some systemic effects.^6^^,^^7^ First, intravesical BCG immunotherapy induces the local activation of antigen-presenting cells, producing pro-inflammatory chemokines and cytokines.^8^^,^^9^ This early immune response precedes the recruitment to the bladder of granulocytes and mononuclear cells, including monocytes, natural killer (NK) cells, and B and T lymphocytes.^10^^,^^11^ In addition, the presence of CD4^+^ T helper type 1 cells (Th1) into the bladder, secreting high levels of IFNγ, TNFα, and IL-2, has been associated with a favorable prognosis in human cohorts,^12^^,^^13^ suggesting an important role of T cells in BCG-mediated antitumoral immunity. This has been demonstrated in experimental models where T cell depletion led to the loss of BCG-associated anti-tumoral effect.^11^ However, antigen specificity of the bladder-infiltrating T cells remains largely unknown.

Given the strong Th1 response observed after BCG immunotherapy, we hypothesized that a subset of these T cells recognizes BCG-derived epitopes, driving their activation and recruitment to the bladder where they may contribute to the local immune response. While this is widely assumed, direct evidence of BCG-specific CD4^+^ T cell infiltration in the human bladder is lacking, and it remains unclear whether these cells can be detected in urine as a surrogate for bladder infiltration. To address this, we examined whether BCG-specific CD4^+^ T cells expand during therapy and can be detected in blood and urine as a proxy for local immune activity. Demonstrating that BCG-specific CD4^+^ T cells are present locally, where they encounter BCG antigens, and that they are functional and antigen-reactive could provide a critical first step toward understanding their potential contribution to the therapeutic response.

In this study, we followed thirty-two patients with NMIBC undergoing BCG immunotherapy, using longitudinal blood and urine samples collected from two cohorts (14 patients providing biobank peripheral blood mononuclear cell [PBMC] samples, and 18 patients providing fresh urine samples). Using a dedicated pool of peptides, covering 150 validated T cell epitopes of the BCG proteome, we evaluated the presence of pre-existing BCG-specific T cells and monitored the effect of BCG immunotherapy on the frequency and functional phenotype of BCG-specific T cells in both blood and urine samples. This study provides novel biological insights into the cellular mechanisms of BCG-induced systemic and local immune response to intravesical BCG immunotherapy for bladder cancer.

## Results

In this study, we included thirty-two patients (30 males and 2 females) with high-risk NMIBC (14 high-grade Ta, 15 high-grade T1, and 9 CIS including 2 isolated CIS) or intermediate-risk NMIBC for one patient (low-grade Ta). Among them, 5 patients had received intravesical BCG induction or maintenance therapy prior to their enrollment and were annotated as BCG-experienced, with 1 also receiving intravesical mitomycin C. Peripheral blood samples were obtained from 14 patients during the BCG induction phase, and urine samples were collected from 18 patients during the first and second maintenance phases, resulting in a total of 58 biological samples (36 blood and 22 urine). Patients were recruited at Saint Louis and Bichat hospitals in Paris where they received BCG immunotherapy. Individual patient and tumor characteristics are available in Table S1, and the contribution of each patient to specific analyses is detailed in Table S2.

### Pre-existing BCG-specific CD4^+^ T cells are detectable in most patients with bladder cancer, prior to BCG immunotherapy

**Figure 1:**
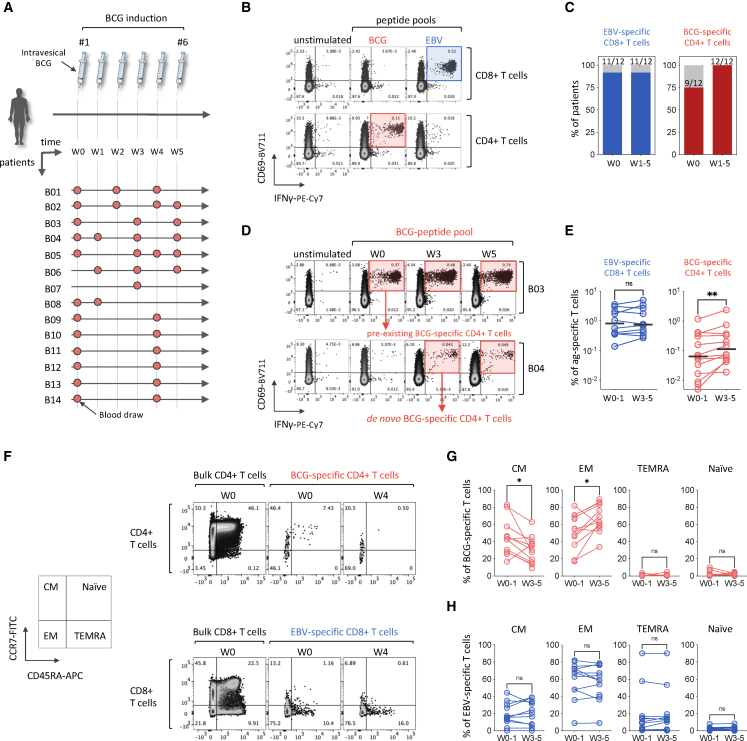
BCG immunotherapy for bladder cancer increases the frequency and promotes memory differentiation of circulating BCG-specific CD4^+^ cells (A) Overview of the BCG immunotherapy phases and timeline, study participants, and blood sample collection. (B) Representative flow cytometry plots of the combined ICS and AIM assay for the detection of antigen-specific CD8^+^ (top) and CD4^+^ (bottom) T cells in the PBMCs of patient B13, four weeks (W4) following BCG immunotherapy induction. Cells were stimulated with BCG- or EBV-peptide pools for 6 h. Frequencies of CD69^+^IFNγ^+^ antigen-specific T cells are indicated. (C) Frequency of patients with detectable EBV-specific CD8^+^ or BCG-specific CD4^+^ T cells at baseline (W0) and up to five weeks (W5) after initiation of BCG immunotherapy. Patients B6 and B7 were excluded from the analysis due to the absence of baseline samples. (D) Flow cytometry plots showing a patient (B3) with pre-existing BCG-specific CD4^+^ T cells (upper) and a patient (B4) with no pre-existing BCG-specific CD4^+^ T cells at baseline but detectable *de novo* BCG-specific CD4^+^ T cells following BCG immunotherapy initiation. (E) Changes in the frequency of circulating EBV-specific CD8^+^ or BCG-specific CD4^+^ T cells between early (W0–W1) and late (W3–W5) time points after initiation of BCG immunotherapy. Statistical significance was tested by first assessing normality of the paired differences (*n* = 11 for EBV-specific CD8^+^ T cells and *n* = 12 for BCG-specific CD4^+^ T cells) using the Shapiro-Wilk test. As the data were non-normally distributed, we used a Wilcoxon signed-rank test. Patients B7 and B8 were excluded due to the absence of samples at early (W0–W1) and late (W3–W5) time points, respectively. Patient B5 showed no detectable EBV-specific T cells at any tested time point. Bars represent the median frequencies of the antigen-specific T cells. (F) Representative flow cytometry plots of memory T cell subsets, based on CD45RA and CCR7 co-expression patterns, in bulk CD4^+^ and BCG-specific CD4^+^ T cells (top), or bulk CD8^+^ and EBV-specific CD8^+^ T cells (bottom). (G) Frequencies of memory T cell subsets among BCG-specific CD4^+^ T cells at baseline (W0) and between three and five weeks (W3–W5) after initiation of BCG immunotherapy. Statistical significance was tested by first assessing normality of the paired differences (*n* = 11) using the Shapiro-Wilk test. As the data were normally distributed, a paired two-tailed Student’s *t* test was applied. Patients B7 and B8 were excluded due to the absence of samples at early (W0–W1) and late (W3–W5) time points, respectively. Additionally, patient B11 was excluded for lacking detectable BCG-specific T cells at baseline. (H) Frequencies of memory T cell subsets among EBV-specific CD8^+^ T cells at baseline (W0) and between three and five weeks (W3–W5) after initiation of BCG immunotherapy. Statistical significance was tested by first assessing normality of the paired differences (*n* = 11) using the Shapiro-Wilk test. As the data were normally distributed, a paired two-tailed Student’s *t* test was applied. Patients B7 and B8 were excluded due to the absence of samples at early (W0–W1) and late (W3–W5) time points, respectively. Additionally, patient B5 was excluded for lacking detectable EBV-specific T cells at any tested time point. Patient IDs are labeled as “B” for blood samples, followed by the individual patient number; *∗p* < 0.05; ∗∗*p* < 0.01; ns, not significant*.*

First, we investigated the presence of BCG-specific T cells in the peripheral blood of fourteen BCG therapy-naive patients undergoing BCG immunotherapy. A schematic of the BCG treatment timeline and blood sample collection is depicted in Figure 1A. We stimulated the PBMCs *ex vivo* with dedicated pools of peptides: one covering 150 immunodominant T cell epitopes of the BCG proteome, and the other targeting 135 immunodominant T cell epitopes of Epstein-Barr virus (EBV) as a physiological positive control for the detection of antigen-specific T cells. We used a combined intracellular cytokine staining (ICS) and activation-induced marker (AIM) assay to identify antigen-reactive specific T cells, using flow cytometry.^14^ Antigen-specific T cells were characterized as activated (CD69^+^) and IFNγ-producing T cells (Figures 1B and S1A). The analysis of the PBMC samples at baseline (W0) revealed that most of the patients (9 out of 12; 75%) had pre-existing BCG-specific CD4^+^ T cells prior to BCG immunotherapy initiation (Figures 1B–1D). This finding aligns with the historically high BCG vaccine coverage in France, where approximately 95% of children were vaccinated by age 6 before the cessation of universal BCG vaccination between 2005 and 2007.^15^ In this study, no BCG-specific CD8^+^ T cells were detected either prior to or after the initiation of BCG immunotherapy. This observation is consistent with prior evidence that BCG vaccination or exposure to *Mycobacterium tuberculosis* predominantly elicits CD4^+^ T cell responses, as opposed to CD8^+^ T cell responses.^16^^,^^17^ In contrast, almost all the patients presented EBV-specific T cell responses dominated by CD8^+^ T cell responses (Figures 1B and 1C).

### BCG immunotherapy increases the frequency and promotes memory differentiation of circulating BCG-specific CD4^+^ Th1 polyfunctional cells

The longitudinal analysis of antigen-specific T cells from baseline (W0) to up to 5 weeks (W5) following primo-BCG immunotherapy showed that three patients without pre-existing BCG-specific T cells (B4, B8, and B11) developed BCG-specific CD4^+^ T cells during the course of BCG immunotherapy (Figures 1C, 1D, and S1B). To evaluate how the frequency of BCG-specific CD4^+^ T cells evolved over time, we compared early (W0–W1) versus late (W3–W5) time points. This comparison revealed that the frequency of BCG-specific CD4^+^ T cells increased by 1.75-fold between W0–W1 and W3/W5, going from a median of 0.064%–0.112% of total CD4^+^ T cells (∗∗*p* = 0.0098), while the frequency of EBV-specific T cells did not significantly change during the treatment (Figure 1D). Given the important size of the BCG proteome, comprising around 3,500 proteins,^18^ the use of a restricted 150-peptide pool—although targeting dominant regions of the BCG proteome—has likely led to an underestimation of the frequency of BCG-specific T cells. While this was not a predefined objective of the study, we compared responses to our 150-peptide BCG pool with a cross-reactive *Mycobacterium tuberculosis* (MTB) peptide pool containing 300 validated epitopes in a subset of three patients (B1, B2, and B5). In all tested samples, stimulation with the MTB pool resulted in consistently higher frequencies of CD69^+^ IFN-γ^+^ CD4^+^ T cells compared with the BCG pool (Figures S1C and S1D). Similarly, a previous study by Elsäßer et al. showed higher frequencies of purified protein derivate (PPD)-specific CD4^+^ T cells, reaching approximatively 0.42% of total CD4^+^ T cells, in the peripheral blood of patients with urothelial carcinoma by W4–W5 following BCG immunotherapy induction.^19^ Nevertheless, the use of larger peptide pool and protein lysates could lead to toxicity or introduce false-positive results due to unspecific stimulation of the cells,^20^ justifying the use of a more restricted BCG-specific peptide pool.

To extend these findings, we investigated whether BCG-specific CD4^+^ T cells undergo memory differentiation during BCG immunotherapy. At early time points (W0–W1), BCG-specific CD4^+^ T cells predominantly exhibited a central memory (CM) phenotype (CCR7^+^ CD45RA^−^), with a median frequency of 42.45%. By weeks 3–5 (W3–W5) post-BCG induction, this proportion significantly decreased to 30.5% (∗*p* = 0.0246) (Figures 1F and 1G). Interestingly, during the same period, the cells shifted toward a dominant effector-memory (EM) phenotype (CCR7^−^ CD45RA^−^), increasing to a median frequency of 62.8% (∗*p* = 0.0175) (Figures 1F and 1G), suggesting a transition from memory to effector T cells. In contrast, no memory differentiation of EBV-specific CD8^+^ T cells was observed during BCG immunotherapy (Figures 1F and 1H).

**Figure 2:**
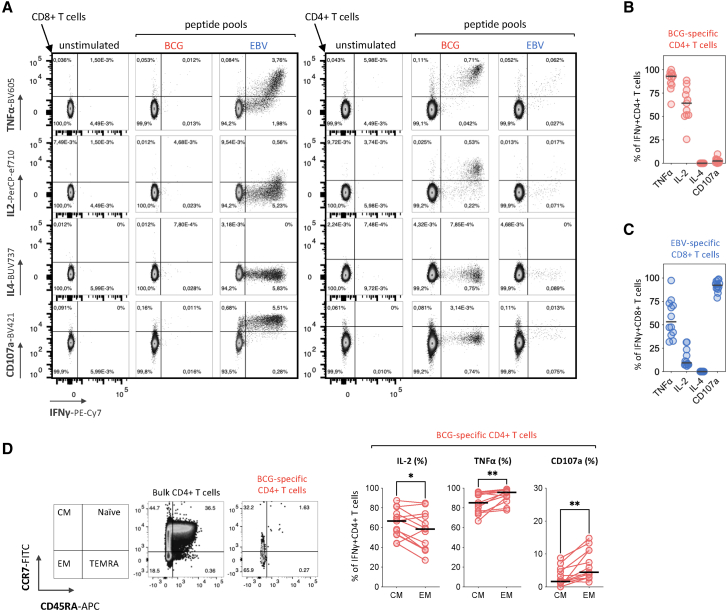
BCG-specific CD4^+^ T cells express a helper type 1 (Th1) polyfunctional phenotype (A) Representative flow cytometry plots of cytokine production and cytotoxic activity of BCG- and EBV- specific T cells, following stimulation with BCG- or EBV-peptide pools. Frequencies of TNFα-, IL-2-, IL-4-, CD107a-, and IFNγ-producing cells are indicated. (B) Dot plot histograms displaying the frequency of BCG-specific CD4^+^ T cells (IFNγ^+^) co-producing TNFα, IL-2, and IL-4 or co-expressing CD107a at W3–W5 post-BCG-induction. Bars represent the median frequencies of the BCG-specific CD4^+^ T cells across patients (*n* = 13). Patients B8 was not included due to the absence of samples at late (W3–W5) time points. (C) Dot plot histograms displaying the frequency of EBV-specific CD8^+^ T cells (IFNγ^+^) co-producing TNFα, IL-2, and IL-4 or co-expressing CD107a at W3–W5 post-BCG-induction. Bars represent the median frequencies of EBV-specific CD8^+^ T cells across the patients (*n* = 12). Patients B5 and B8 were excluded due to absence of detectable EBV-specific CD8^+^ T cells and lack of samples at late (W3–W5) time points, respectively. (D) Representative flow cytometry plots of the gating strategy to isolate central memory (CM) and effector memory (EM) BCG-specific CD4^+^ T cells (left) and dot plot histograms displaying the frequency of BCG-specific CD4^+^ T cells (IFNγ^+^) co-producing IL-2 and TNFα or co-expressing CD107a depending on the memory compartment (CM or EM; right). Statistical significance was tested by first assessing normality of the paired differences using the Shapiro-Wilk test. As the data were normally distributed, a paired two-tailed Student’s *t* test was applied. ∗*p* < 0.05; ∗∗*p* < 0.01.

Next, we investigated the functional phenotype of BCG-specific CD4^+^ T cells by ICS and multiparametric flow cytometry, after stimulation with BCG-peptide pool (Figure 2A). Here, we focused on BCG-specific T cells at week 4 (W3–W5) post-BCG immunotherapy induction in order to capture the functional phenotype of BCG-induced effector T cells. This analysis revealed that a majority of BCG-specific CD4^+^ T cells expressing IFNγ also expressed TNFα (92.98%) and IL-2 (64.2%). At the same time, no IL-4 and low frequencies of CD107a-expressing BCG-specific CD4^+^ T cells were detected, suggesting a dominance of Th1 responses (Figures 2A and 2B). EBV-specific CD8^+^ T cells, used as a control, presented a functional phenotype dominated by IFNγ and CD107a co-expressing T cells (91.1%), consistent with degranulation and potential cytotoxic function (Figures 2A and 2C).

To further investigate the functional profile of BCG-specific CD4^+^ T cells during therapy, we compared the frequency of BCG-specific CD4^+^ T cells expressing functional markers (IL-2, TNFα, and CD107a) within the CM (CCR7^+^CD45RA^−^) and effector memory (EM; CCR7^−^CD45RA^−^) subsets. As shown in Figure 2D, IL-2-producing cells were more frequent within the CM subset (66.7%) as compared with the EM subset (58.5%; ∗*p* = 0.0251), whereas TNFα^+^ cells were more enriched within the EM subset (95.6%), as compared with the CM subset (85.2%; ∗∗*p* = 0.0090). Although CD107a^+^ cells represented only a small fraction overall (less than 10%), their frequency was higher within the EM subset (4.4%) than within the CM subset (1.64%; ∗∗*p* = 0.0016), suggesting the acquisition of degranulation capacity for at least a small proportion of circulating BCG-specific CD4^+^ T cells.

These findings indicate a functional diversification of circulating BCG-specific CD4^+^ T cells during immunotherapy, with effector-associated functions emerging alongside IL-2-producing memory responses. Altogether, the data show that BCG immunotherapy in patients with bladder cancer induces the expansion and functional maturation of circulating BCG-specific CD4^+^ T cells to a Th1 polyfunctional profile.

### BCG immunotherapy triggers the local recruitment of BCG-specific CD4^+^ Th1 cells to the bladder

**Figure 3:**
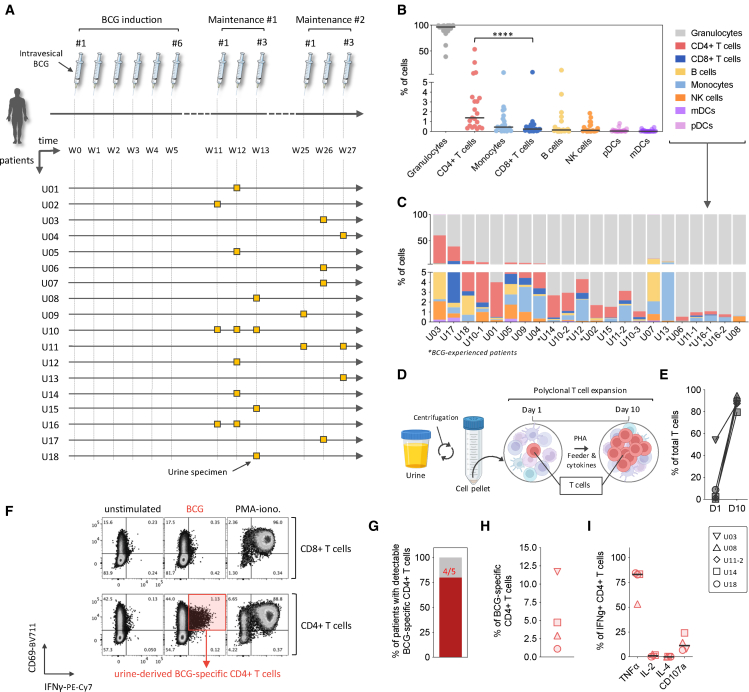
Intravesical BCG immunotherapy induces the local recruitment of BCG-specific CD4^+^ Th1 cells (A) Overview of the BCG-therapy phases and timeline, study participants, and urine specimen collection. (B) Frequency of the different immune cells in urine samples, as identified by flow cytometry analysis performed *ex vivo*. Statistical analysis of the T cell subset proportions was performed using Wilcoxon tests (paired, nonparametric, two-sided) on urine samples (*n* = 20). Bars represent the median frequencies of the specified cell types. Statistical significance was tested by first assessing normality of the paired differences (frequency of CD4^+^ and CD8^+^ T cells) using the Shapiro-Wilk test. As the data were not normally distributed, we used a Wilcoxon signed-rank test. ∗∗∗∗*p* < 0.0001. (C) Distribution of different immune cells across the urine samples, *ex vivo*. (D) Schematic of the polyclonal T cell expansion protocol from urine samples. (E) Frequency of T cells in five urine samples before and 10 days after polyclonal T cell expansion. (F) Representative flow cytometry plots of BCG-specific CD4^+^ T cells in the urine of patient U18, at week 13 (W13) of BCG immunotherapy, following polyclonal T cell expansion and 6-h stimulation with the BCG-peptide pool. Frequency of cells is indicated. (G) Frequency of patients with detectable BCG-specific CD4^+^ T cells in the urine following polyclonal T cell expansion. (H) Frequency of BCG-specific CD4^+^ T cells in the urine samples of patients (*n* = 4), following polyclonal T cell expansion and 6-h stimulation with the BCG-peptide pool. (I) Dot plot histograms displaying the frequency of BCG-specific CD4^+^ T cells (IFNγ^+^) co-producing TNFα, IL-2, and IL-4 or co-expressing CD107a, following polyclonal T cell expansion of urine cells and restimulation with the BCG-peptide pool. Bars represent median frequencies of the BCG-specific CD4^+^ T cells across the patients (*n* = 4). Patient IDs are labeled as “U” for urine samples, followed by the individual patient number.

Studies in mouse models have shown the presence of BCG antigens in bladder-draining lymph nodes of BCG-treated mice,^21^ supporting the possible development of both systemic and local BCG-specific T cell responses. To explore the local recruitment of BCG-specific T cells to the bladder of patients, we analyzed urine specimens as a proxy for bladder-infiltrating immune cells.^10^^,^^22^^,^^23^^,^^24^^,^^25^ Eighteen patients were recruited, and twenty-two urine samples were collected during the first and second maintenance phases of BCG immunotherapy, including three patients with two to three longitudinal urine specimens collected (Figure 3A and Table S1). The rationale for collecting urine specimens after the induction phase, during the first and second maintenance phases of BCG immunotherapy, was that repeated BCG instillations during maintenance would elicit a more robust and sustained T cell immune response. This timing was chosen to facilitate the detection of BCG-specific T cells activated and recruited to the bladder following prolonged antigen exposure.

*Ex vivo* immunophenotyping analysis of urine cells by flow cytometry revealed, as expected, a high frequency of granulocytes, with a median proportion of 96.79% (Figures 3B and S2A), indicative of an ongoing inflammatory reaction. Other immune cell types detected among urine cells, ranked by median frequencies, included CD4^+^ T cells (1.61%), monocytes (0.42%), CD8^+^ T cells (0.24%), B cells (0.15%), NK cells (0.1%), plasmacytoid dendritic cells (pDCs) (0.04%), and myeloid dendritic cells (mDCs) (0.01%) (Figure 3B), despite important variability in the total number (Figure S2B) and immune cell composition (Figure 3C) of the urine cells across the patients. Among T lymphocytes, CD4^+^ T cells were present at a notably higher frequency than CD8^+^ T cells (∗∗∗∗*p* < 0.0001) (Figure 3B). These findings align with prior studies reporting similar frequencies of leukocyte subsets^10^^,^^25^^,^^26^ and elevated CD4^+^ T cell frequency over CD8^+^ T cell frequency in bladder- and tumor-infiltrating lymphocytes following BCG immunotherapy.^21^^,^^27^^,^^28^^,^^29^ To assess whether prior BCG therapy influenced the immune cell composition, we compared the urine samples from BCG-therapy-naïve (*n* = 16) and BCG-experienced (*n* = 6, including two samples from one individual) patients (Figure S2C). Urine from BCG-experienced patients contained overall fewer immune cells, with significantly lower frequencies of CD8^+^ T cells (∗*p* = 0.0243), B cells (∗*p* = 0.0177), mDCs (∗*p* = 0.0351), and pDCs (∗∗*p* = 0.0090) compared with those in BCG-naïve samples, while differences in CD4^+^ T cell frequencies were not significant.

Given the sparse information about antigenic specificity of the T cells infiltrating the bladder during BCG immunotherapy, we intended to detect and evaluate the proportion of BCG-specific T cells in the urine of bladder cancer patients receiving BCG immunotherapy. Because urine typically contains very low numbers of lymphocytes (on average ∼25,000 total cells/mL, see Figure S2B), we did not attempt to measure BCG-specific responses directly *ex vivo*. Instead, we first applied a polyclonal expansion of total urine T cells (Figures 3D and S2D), allowing a downstream analysis of antigen-specific T cells upon antigenic stimulation.^27^ Because urine contained very few lymphocytes, only a subset of samples yielded enough viable T cells for expansion and downstream functional analysis. In these five cases, the expansion step resulted in a median 66.36-fold increase in T cells, ranging from 13.384% to 87.86% of the total live cells (Figure 3E). Notably, stimulation of the expanded T cells with a BCG-peptide pool revealed the presence of BCG-specific CD4^+^ T cell responses in 4 out of 5 patients (80%) tested. In contrast, no BCG-specific CD8^+^ T cells were detected (Figures 3F, 3G, and S2E). The frequency of BCG-specific CD4^+^ T cells ranged from 1% to up to 11.24% of the total CD4^+^ T cells (Figure 3H), providing the baseline evidence for BCG-specific CD4^+^ Th1 cell recruitment to the bladder. This finding highlights T cell trafficking in response to BCG and reveals important inter-individual variations in BCG-specific T cell levels in urine. Urinary BCG-specific CD4^+^ T cells exhibited a Th1 cytokine profile similar to those in peripheral blood, with a strong co-production of IFNγ and TNFα (median 83%; *n* = 4) (Figures 3I and S2E). However, unlike blood-derived cells, urinary BCG-specific CD4^+^ T cells showed minimal IL-2 production (median 1.015%; *n* = 4) but included a small fraction that co-expressed CD107a (median 11.24%; *n* = 4), consistent with degranulation and potential cytotoxic function. These differences may, however, result from the T cell polyclonal expansion step (Figures 2B and 3I). Altogether, these data provide the first line of evidence that BCG-specific CD4^+^ Th1 cells are locally recruited to the bladder of patients receiving intravesical instillations of BCG immunotherapy.

## Discussion

This study provides strong evidence that BCG immunotherapy elicits systemic and local immune responses in NMIBC patients, specifically by increasing the frequency of BCG-specific CD4^+^ Th1 cells in both peripheral blood and the bladder. Given the extensive size of the BCG proteome,^18^ the use of a limited BCG-specific peptide pool (150 peptides) likely underestimated the frequency of BCG-specific T cells detected. Therefore, the actual frequencies of BCG-specific T cells *in vivo* are likely higher than those observed experimentally, reinforcing the idea that BCG immunotherapy induces a more robust immune response than currently measurable.

The presence of pre-existing BCG-specific CD4^+^ T cells in a majority of patients before therapy initiation suggests prior exposure to BCG vaccine and reveals a reservoir of memory T cells, which may influence the immune response to BCG treatment. It should be noted, however, that the peptide pool used in this study, although composed of validated epitopes described in BCG, contains sequences that are also conserved within the *M. tuberculosis* complex and possibly with non-tuberculous mycobacteria. Therefore, baseline responses could, in part, reflect prior exposure to other mycobacteria. We, nevertheless, refer to these responses as “BCG-specific,” since the epitopes tested are present in the BCG proteome used for intravesical therapy.

In line with previous observations in NMIBC patients receiving intravesical BCG,^19^ our data confirm that BCG immunotherapy induces systemic functional activation of CD4^+^ T cells characterized by Th1 cytokine production. Beyond this global activation, our results provide evidence for the functional diversification of BCG-specific CD4^+^ T cells during therapy, with IL-2-producing CM cells coexisting with effector memory cells enriched for TNFα and some expressing CD107a. This pattern suggests progressive effector differentiation of antigen-experienced T cells while maintaining a pool of memory cells. Together, these findings support a model in which intravesical BCG not only expands BCG-specific T cells but also shapes their functional heterogeneity.

Pre-existing immunity to BCG has been associated with increased T cell infiltration in the bladder,^21^ but a causal link between BCG-specific immunity and tumor clearance following BCG immunotherapy has not been established. Although the number of BCG therapy-experienced samples was limited in our study, the reduced immune cell frequencies observed in these patients may reflect altered bladder immune dynamics with prior BCG exposure. However, because the BCG-experienced status may indicate either prior immune stimulation or disease recurrence following suboptimal response to initial therapy, it is unclear whether this profile represents immune adaptation, exhaustion, or features of non-responders. Nevertheless, the role and clinical implication of bladder-infiltrating, BCG-specific T cells remain largely unknown. Additional prospective studies on larger cohorts of patients are necessary to better understand whether the level and phenotype of BCG-specific T cells infiltrating the bladder and detectable in the urine may have clinical implications for the response to therapy and survival.

An additional limitation of this pilot study is that peripheral blood and urine samples were analyzed in separate settings, using biobanked PBMCs from the induction phase and fresh urine samples from the maintenance phase. This design prevented direct paired comparisons of systemic and local responses within the same patients. Future studies will prospectively analyze fresh paired blood and urine samples to enable such comparisons.

While technically challenging, due to the varying and usually low number of lymphocytes in urine (with most cells being granulocytes), it was not feasible in this study to assess BCG-specific responses directly *ex vivo*. We, therefore, relied on a polyclonal expansion protocol to amplify the T cell population present in urine samples, followed by peptide restimulation to reveal antigen-specific reactivity. This strategy enabled us to detect BCG-specific CD4^+^ T cells in 4 out of 5 expanded urine samples, demonstrating feasibility of their detection in this compartment. Future studies are needed to characterize urinary BCG-specific T cells, directly *ex vivo*, without prior expansion step, to more accurately define their abundance and functional status. Indeed, while the polyclonal expansion of T cells from urine samples enabled the detection of BCG-specific T cells, this experimental setup may have altered the cells’ phenotype, limiting the ability to accurately assess their *in vivo* characteristics. Previous studies in NMIBC treated with BCG immunotherapy have suggested that treatment failure may be linked to T cell exhaustion.^23^^,^^30^^,^^31^ Thus, a longitudinal analysis of the functional phenotype of urinary BCG-specific T cells, investigating changes in cytokine secretion and in the expression of inhibitory receptors (i.e., PD-1, CTLA-4, Lag-3, etc.) will be very informative to understand the following: (1) the mechanisms of action of BCG-specific T cells and their potential antitumoral role, and (2) whether these mechanisms may be altered over time in some patients. Immune checkpoint inhibitors (ICIs), such as anti-PD-1 antibodies, are now recommended for advanced urothelial carcinomas.^3^ Consequently, several clinical trials, including ALBAN,^32^ CREST,^33^ and POTOMAC,^34^ are investigating the clinical efficacy of combining ICIs, such as anti-PD-1 or anti-PDL-1 antibodies, with BCG immunotherapy compared to BCG treatment alone. Given the positive results of the CREST study,^35^ it will be important in the future to investigate the effect of ICIs on the breadth and magnitude as well as the functional phenotype of BCG-specific T cells in both peripheral blood and bladder compartments.

In conclusion, this study demonstrates that BCG immunotherapy not only increases circulating BCG-specific CD4^+^ T cell frequencies but also promotes the recruitment of BCG-specific CD4^+^ Th1 cells to the bladder of patients. By demonstrating that BCG-specific CD4^+^ T cells are detectable in urine during therapy and retain functional reactivity, our study provides primary evidence that such cells are present at the site of antigen exposure and may contribute to the local immune response. Furthermore, this study shows that detecting and monitoring BCG-specific T cells in urine samples from bladder cancer patients undergoing BCG therapy are technically feasible. This could provide a valuable cellular and functional readout for assessing the impact of checkpoint inhibitor combination therapies on the level of antigen-specific T cells detected in the urine or for evaluating the prognostic value of urinary BCG-specific T cells. Overall, these findings lay the groundwork for further research into the anti-tumor role and prognostic potential of BCG-specific CD4^+^ T cells detected in urine, ultimately guiding future therapeutic strategies to improve patient outcomes.

### Limitations of the study

The main limitations of this study include the restricted breadth of the BCG-specific peptide pool used, which likely underestimated the true frequency of BCG-specific T cells. Pre-existing responses may also reflect prior exposure to other mycobacteria, given epitope conservation across the *Mycobacterium tuberculosis* complex and some non-tuberculous species. Additionally, peripheral blood and urine samples were collected and analyzed in different clinical phases, preventing a direct paired assessment of systemic and local immunity within the same patients. The detection of urinary BCG-specific T cells required a polyclonal expansion step due to typically low lymphocyte yields in urine, a process that may alter the cellular phenotype and limit the accuracy of *in vivo* characterization. Finally, the limited numbers of BCG-experienced patients and urine samples constrain the generalizability of the findings and underscores the need for larger, prospective studies to more fully delineate the abundance, phenotype, and clinical relevance of BCG-specific T cells in the bladder and urine.

## Resource availability

### Lead contact

Further information and requests for resources and reagents should be directed to and will be fulfilled by the lead contact, Dr. Pierre Tonnerre (pierre.tonnerre@inserm.fr).

### Materials availability

This study did not generate new unique reagents. All other data are available upon request from the lead contact.

### Data and code availability

• The published article includes all data generated or analyzed during this study, which are summarized in the accompanying figures and supplemental information.
• This study did not generate custom code.
• Any additional information required to reanalyze the data reported in this work paper is available from the lead contact upon request.

## Acknowledgments

This work was supported by the 10.13039/501100006431Cancéropôle Île-de-France (2022-1-EMERG-07-INSERM_7-1), the 10.13039/501100004097Fondation ARC pour la Recherche sur le Cancer (Sys-Can ARCPGA12021 020003101_358 7), and the Laboratory of Excellence (Labex) 10.13039/100018737Inflamex. We thank the Institut de Recherche Saint-Louis flow cytometry facility, in particular Niclas Setterblad, Claire Maillard, and Christelle Doliger, for their technical support, as well as Maxime Frelaut, Adeline Uko, and Sylia Mezred for their help in biological sample collection. P.R. received a fellowship from the 10.13039/501100016396Association Française d'Urologie. M.B. received a fellowship from La Ligue Contre le Cancer. P.T.’s research activity is supported by the ATIP-Avenir program from 10.13039/501100001677Inserm and CNRS.

## Author contributions

P.R. and B.P. built and managed the circuit of samples, performed the experiments, analyzed flow cytometry data, and wrote the manuscript; E.A., T.L.V., D.K.-E., D.B., M.B., and F.O. performed the experiments and contributed to the data analysis; J.L.M., N.R.-F., A.M-L., and F.D. facilitated access to biological samples and clinical information and contributed to the study design and data interpretation; M.F.C., C.S.L.A., and A.S. contributed to the study design and data interpretation; E.X. and P.T. conceived the project, provided funding, and supervised the work. All authors reviewed the manuscript.

## Declaration of interests

All authors declare no competing interests.

## STAR★Methods

### Key resources table

**Table:** 

REAGENT or RESOURCE	SOURCE	IDENTIFIER
**Antibodies**		

Anti-human CD3 AF700 (clone UCHT1)	BioLegend	cat# 300424; RRID: AB_493741
Anti-human CD4 BV421 (clone OTK4)	BioLegend	cat# 317434; RRID: AB_2562134
Anti-human CD8 PE-Dazzle 594 (clone SK1)	BioLegend	cat# 344744; RRID: AB_2566515
Anti-human CD11c APC (clone B-ly6)	BD Biosciences	cat# 559877; RRID: AB_398680
Anti-human CD14 BUV737 (clone M5E2)	BD Biosciences	cat# 612763; RRID: AB_2870094
Anti-human CD15 PerCP-eFluor710 (clone MMA)	Invitrogen	cat# 46-0158-41; RRID: AB_10804276
Anti-human CD16 PE (clone 3G8)	BD Biosciences	cat# 556619; RRID: AB_396491
Anti-human CD19 FITC (clone REA675)	Miltenyi Biotec	cat# 130-113-645; RRID: AB_2726198
Anti-human CD45 APC-Cy7 (clone 2D1)	BD Biosciences	cat# 557833; RRID: AB_396891
Anti-human CD56 BUV395 (clone NCAM16.2)	BD Biosciences	cat# 563554; RRID: AB_2687886
Anti-human CD123 BV650 (clone 6H6)	BioLegend	cat# 306020; RRID: AB_2563827
Anti-human HLA-DR BV711 (clone L243)	BioLegend	cat# 307644; RRID: AB_2562913
Anti-human CD19 BV510 (clone HIB19)	BioLegend	cat# 302242; RRID: AB_2561668
Anti-human CD4 APC-Cy7 (clone RPA-T4)	BD Biosciences	cat# 557871; RRID: AB_396913
Anti-human CD8 BUV395 (clone RPA-T8)	BD Biosciences	cat# 563796; RRID: AB_2722501
Anti-human CCR7 APC (clone G043H7)	BioLegend	cat# 353214; RRID: AB_10917387
Anti-human CD45RA FITC (clone HI100)	BioLegend	cat# 304106; RRID: AB_314410
Anti-human CD69 BV711 (clone FN50)	BioLegend	cat# 310944; RRID: AB_2566466
Anti-human CD137 Pe-Dazzle 594 (clone 4B4-1)	BioLegend	cat# 309826; RRID: AB_2566260
Anti-human CD107a BV421 (clone H4A3)	BioLegend	cat# 328626; RRID: AB_10899581
Anti-human IFNɣ Pe-Cy7 (clone 4S.B3)	Invitrogen	cat# 25-7319-82; RRID: AB_469682
Anti-human TNFɑ BV605 (MAb11)	BioLegend	cat# 502936; RRID: AB_2563884
Anti-human IL-2 PerCP-eFluor710 (clone MQ1-17H12)	Invitrogen	cat# 46-7029-42; RRID: AB_1834419
Anti-human IL-4 BUV737 (clone MP4-25D2)	BD Biosciences	cat# 612835; RRID: AB_2870157

**Biological samples**		

Urine cells of patients undergoing BCG-therapy	AP-HP, Hôpital Bichât & Saint-Louis	–
PBMC from patients undergoing BCG-therapy	AP-HP, Hôpital Bichât & Saint-Louis	–
50 Gy irradiated allogeneic PBMC	Etablissement Français du Sang (EFS)	–

**Chemicals, peptides and recombinants peptides**		

PepMix Pan-Tuberculosis BCG Select	JPT	PM-Pan-TBBCGselect-2
PepMix™ Pan-EBV Select	JPT	PM-Pan-EBVselect-1
FastImmune™ Co-Stimulatory Antibodies (CD28/CD49d)	BD Biosciences	cat# 347690
Protein Transport Inhibitor	Invitrogen	cat# 00-4980-93
Phytohemagglutinin (PHA)	Invitrogen	cat# 00-4977-93
Interleukin-2	Stemcell Technologies	cat# 78220.2
Interleukin-7	Stemcell Technologies	cat# 78053
Interleukin-15	Stemcell Technologies	cat# 78031
Zombie Aqua™ Fixable Viability Kit	BioLegend	cat# 423102

**Software and algorithms**		

FlowJo v10	BD Biosciences	www.flowjo.com
GraphPad Prism v9	GraphPad Software	https://www.graphpad.com

### Experimental model and study participant details

#### Study participants and biological sample collection

Blood and urine samples were collected from patients with bladder cancer undergoing BCG therapy at Saint-Louis and Bichat Hospitals. The study was approved by the “Comité d’Evaluation de l’Ethique des projets de Recherche Biomédicale (CEERB) Paris Nord” Institutional Review Board (IRB 00006477- of HUPNVS Université Paris, AP-HP), and all samples were obtained in accordance with the principles of Good Clinical Practice and the Declaration of Helsinki. Each patient signed an informed consent before inclusion to the study. Demographic and clinical information of patients included in this study are provided in Table S1.

In total, we included thirty-two patients (30 males and 2 females) with high risk NMIBC (14 high grade Ta, 15 high grade T1, and 9 CIS including 2 isolated CIS), or with intermediate risk NMIBC for one patient (low grade Ta). Among them, 5 patients had received prior intravesical BCG induction or maintenance therapy before study enrollment and were annotated as BCG-experienced, with 1 also receiving intravesical mitomycin C. Peripheral blood samples were obtained from 14 patients during the BCG induction phase (Figure 1A), and urine samples were collected from 18 patients during the first and second maintenance phases (Figure 3A), at the time of bladder catheterization (prior to BCG instillation), resulting in a total of 58 biological samples (36 blood and 22 urine).

All patients included in this study had negative cytobacteriological examination results, which were systematically performed alongside clinical instillations. Due to the low number of study participants, the influence of age and sex could not be assessed.

### Method details

#### Blood and urine processing

Peripheral blood mononuclear cells (PBMCs) were isolated from patient’s peripheral blood samples collected prior to BCG instillations using a Lymphoprep (Stemcell) density gradient, washed in PBS, frozen in a 90% FBS (Eurobio) and 10% DMSO (Sigma) solution, and stored in liquid nitrogen. After thawing, PBMCs were treated with DNase I (Roche) in RPMI + Glutamax (Gibco) supplemented with 10% FBS (Eurobio), 1% MEM NEAA (Gibco), 1% sodium pyruvate (Gibco), and 1% penicillin/streptomycin (10,000 U/mL, Life Technologies). Cells were rested overnight at 37°C with 5% CO_2_ before further testing.

Urine samples were processed immediately by adding 10% FBS. Urine samples were then centrifuged and cells were collected, filtered using a 70 μm filter, and counted. Around 50,000 cells were immediately stained for an *ex vivo* immunophenotyping by flow cytometry, while the remaining cells were used for polyclonal T cell expansion.

#### Polyclonal T cell expansion from urine samples

Urine cells were mixed with irradiated (50 Gy) allogeneic PBMCs as feeders, at a concentration of 1 million cells per mL. Cells were incubated in U-bottom 96-well plates with RPMI medium containing IL-2, IL-7, and IL-15 at 10 UI/mL along with phytohemagglutinin (PHA), at 2.5 μg/mL. Cultures were expanded over 10–14 days, with fresh medium added every 2–3 days.

#### Cell stimulation for antigen-specific T cells detection

To identify BCG-specific T cells, 2 to 5 million rested PBMCs or expanded T cells from urine were stimulated in supplemented culture media with peptide pools (except for the unstimulated condition) of BCG (JPT PepMix Pan-Tuberculosis BCG Select), EBV (JPT PepMix Pan-EBV Select) or MTB megapool,^20^^,^^36^ at 1 μg/mL and in the presence of 10 μL/mL of anti-CD28/49days antibodies (Fast Immune BD Biosciences), 4 μL of anti-CD107a BV421 (clone H4A3, BioLegend) antibodies, at a final volume of 400 μL. An unstimulated condition containing anti-CD28 alone was used as a negative control, while a 1X Cell Stimulation Cocktail of PMA and ionomycin (eBioscience) served as a positive control. Cells were incubated for 6h at 37 °C and 1X protein transport inhibitor (eBioscience) was added after the first 2h of incubation to facilitate intracellular cytokine detection and quantification.

#### Flow cytometry analysis

For *ex vivo* immunophenotyping of urine leukocytes, dead cells were stained with the Zombie Aqua Fixable Viability Kit (BioLegend) at 4°C, for 15 min, 200uL final. Cells were washed and cells were stained for surface markers at 4°C for 30 min in FACS buffer (PBS 2 mM EDTA and 1% human serum), including the following mix of antibodies; CD3 Alexa Fluor 700, CD4 Brilliant Violet 421, CD8 PE-Dazzle 594, CD11c APC, CD14 Brilliant Ultra Violet 737, CD15 PerCP-eFluor 710, CD16 PE, CD19 FITC, CD45 APC-Cy7, CD56 Brilliant Ultra Violet 395, CD123 Brilliant Violet 650, and HLA-DR Brilliant Violet 711. Cells were washed and fixed with 2% paraformaldehyde until further analysis by flow cytometry.

For antigen-specific detection by intracellular staining, stimulated PBMCs or expanded T cells from urine were stained for dead cells and surface antibodies, including CD19 Brilliant Violet 510, CD3 Alexa Fluor 700, CD4 APC-Cy7, CD8 Brilliant Ultra Violet 395, CCR7 APC, CD45RA FITC, CD69 Brilliant Violet 711, CD137 PE-Dazzle 594, as described above. Cells were washed then fixed and permeabilized in a “Fix/Perm” solution (Invitrogen, 00-5123-43) at room temperature for 30 min. Cells were then stained with a mix of antibodies targeting intracellular cytokines, including IFNɣ Pe-Cy7, TNFɑ Brilliant Violet 605, IL-2 PerCP-eFluor 710, and IL-4 Brilliant Ultra Violet 737, at 4°C, for 30 min in permeabilization buffer (Invitrogen, 00-5123-43). Cells were washed, resuspended in PBS and analyzed using a BD LSR II FORTESSA flow cytometer.

### Quantification and statistical analysis

#### Data analysis and identification of antigen-specific T cells

Flow cytometry data were analyzed using FlowJo software. Protein expression levels were extracted as percentages. Bar-plots comparing the frequency of patients with detectable antigen-specific T cells, and dot-plot histograms comparing the expression levels of the proteins across patients, timepoints and stimulation conditions were generated using GraphPad Prism software. Following antigen stimulation with a given peptide pool, antigen-specific T cells were identified by intracellular cytokine staining of IFNɣ and co-expression with the activation marker CD69.^14^ A positive detection was defined as the presence of at least 50 antigen-specific T cells and a frequency at least twice that observed in the matched negative control condition (anti-CD28 alone, without peptide pool).

#### Statistical analysis

Statistical analyses were performed using GraphPad Prism software. Data distributions were assessed with the Shapiro–Wilk test and visual inspection of Q–Q plots. When data were not normally distributed, nonparametric tests were applied. Specifically, paired two-sided Wilcoxon signed-rank tests were used for within-subject comparisons (e.g., early versus late timepoints), and unpaired two-sided Mann–Whitney U tests were used for independent group comparisons (e.g., BCG-naïve versus BCG-experienced samples). When data followed a normal distribution, paired two-sided t-tests were used. The statistical test used and the measure of central tendency are indicated in each figure legend. A *p*-value of less than 0.05 was considered statistically significant (∗*p* < 0.05; ∗∗*p* < 0.01; ∗∗∗*p* < 0.001; ∗∗∗∗*p* < 0.0001).
