# Supplementary material for: BCG immunotherapy for bladder cancer triggers systemic and local BCG-specific CD4+ Th1 responses
Source: iScience. 2026 Jan 12;29(2):114676. doi: 10.1016/j.isci.2026.114676 (PMC12877854; doi:10.1016/j.isci.2026.114676)
Supplement: Document S1. Figures S1 and S2 and Tables S1 and S2 [file mmc1.pdf]

iScience, Volume *29*

Supplemental information

BCG immunotherapy for bladder cancer triggers systemic and local BCG-specific $CD4^+$ Th1 responses

Paul Rollin, Benjamin Pluskwa, Emilie Artru, Tristan Le Vaslot, Daria Kartasheva-Ebertz, Diane Biron, Margaux Bossis, Fanny Onodi, Joel LeMaoult, Nathalie Rouas-Freiss, Mathieu F. Chevalier, Cecilia S. Lindestam Arlehamn, Alessandro Sette, Alexandra Masson-Lecomte, François Desgrandchamps, Evanguelos Xylinas, and Pierre Tonnerre

Figure S1

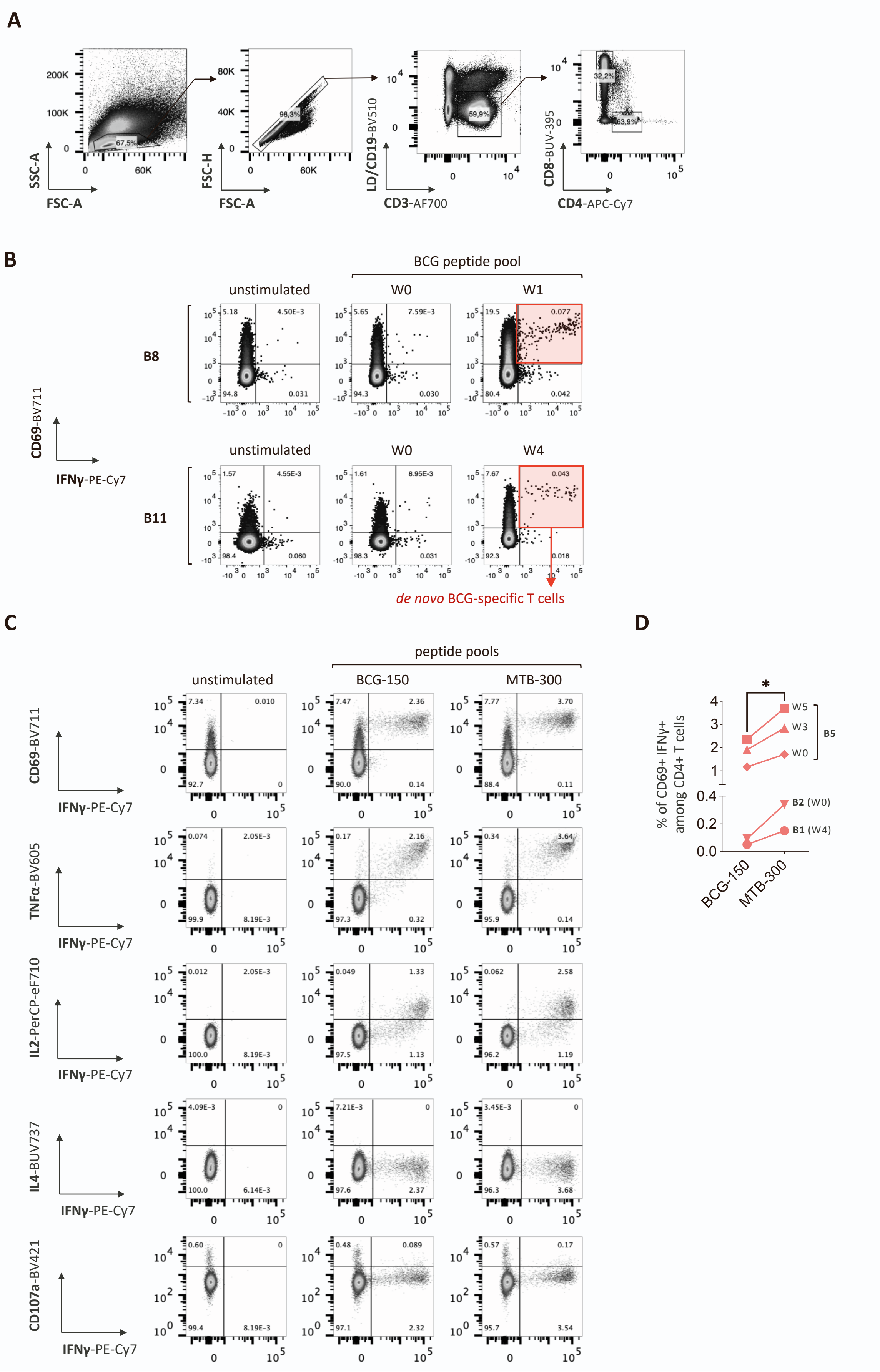
A
SSC-A
FSC-A
FSC-H
FSC-A
LD/CD19-BV510
CD3-AF700
CD8-BUV-395
CD4-APC-Cy7
67,5%
98,3%
59,9%
32,2%
63,9%
B
BCG peptide pool
unstimulated
W0
W1
W4
B8
B11
CD69-BV711
IFNγ-PE-Cy7
de novo BCG-specific T cells
C
peptide pools
unstimulated
BCG-150
MTB-300
CD69-BV711
TNFα-BV605
IL2-PerCP-eF710
IL4-BUV737
CD107a-BV421
IFNγ-PE-Cy7
D
% of CD69+ IFNγ+ among CD4+ T cells
W5
W3
W0
B5
B2 (W0)
B1 (W4)
BCG-150
MTB-300

Figure S1: Gating strategy and representative flow cytometry plots of BCG-specific CD4+ T cells in the peripheral blood of patients with bladder cancer undergoing BCG therapy, related to Figure 1 and 2.

A. Representative flow cytometry gating strategy to isolate CD4+ and CD8+ T cells for downstream analysis of antigen-specific T cells.

B. Longitudinal analysis of BCG-specific CD4+ T cells in the peripheral blood of patients with no detectable pre-existing BCG specific T cells at baseline. Pre-gated on CD4+ T cells.

C. Detection of BCG-specific CD4+ T cells in the peripheral blood of patient B5, 4 weeks (W4) post-BCG induction. PBMC were stimulated with a BCG-peptide pool or with a BCG-cross-reactive Mycobacterium Tuberculosis (MTB)-peptide pool for 6h. Pre-gated on CD4+ T cells.

D. Comparison of CD69+ IFN-γ+ CD4+ T cell responses following stimulation with BCG and MTB peptide pools in a subset of NMIBC patients (patients B1, B2 (single time point), and B5 (three time points)). Statistical significance was tested by first assessing normality of the paired differences using the Shapiro-Wilk test. As the data were normally distributed, a paired two-tailed Student's t-test was applied. Statistical significance was defined as $*p < 0.05$.

Patient IDs are labeled as 'B' for blood samples, followed by the individual patient number.

Figure S2

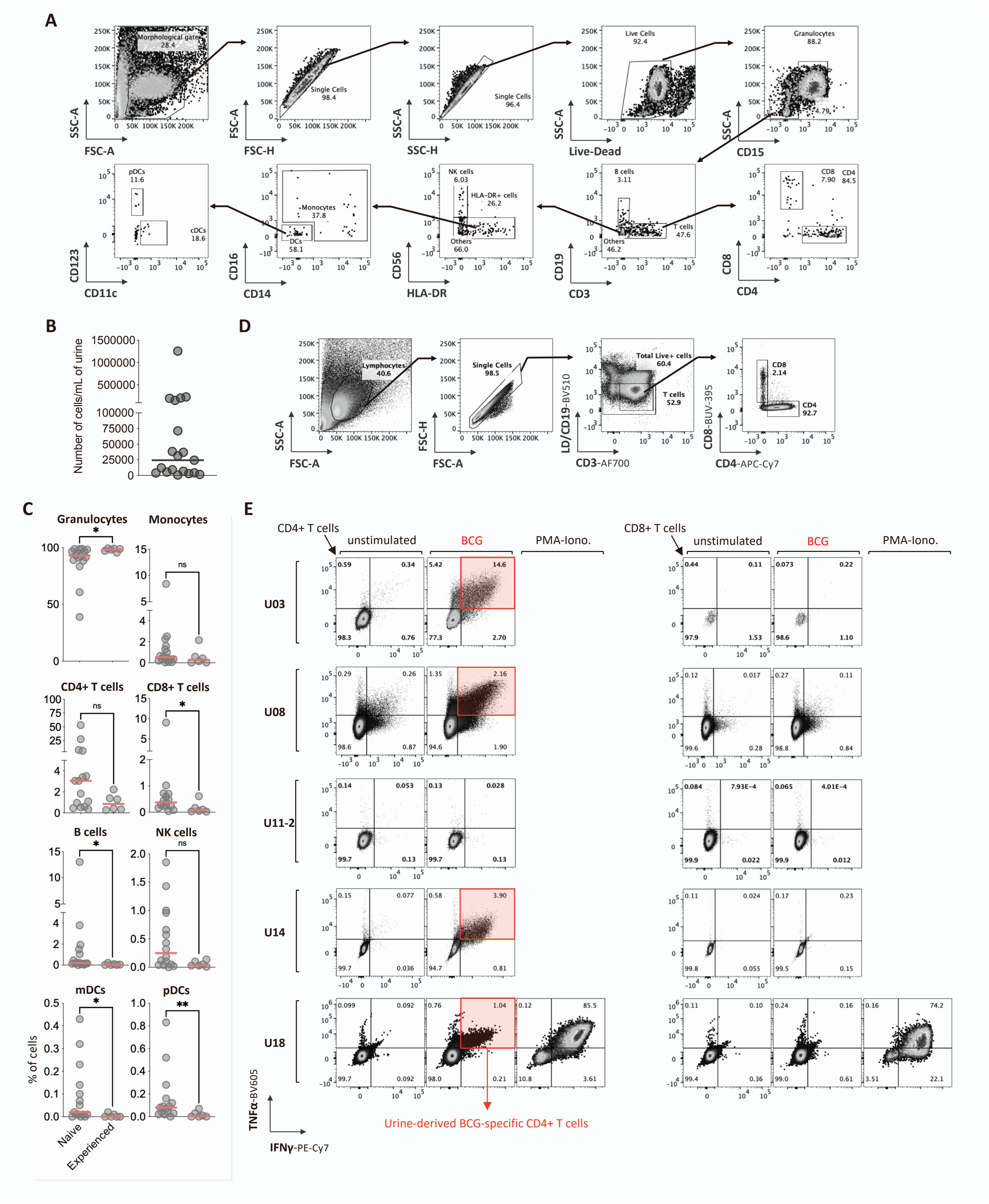
A
Morphological gate 28.4
Single Cells 98.4
Single Cells 96.4
Live Cells 92.4
Granulocytes 88.2
SSC-A
FSC-A
FSC-H
SSC-H
Live-Dead
CD15
pDCs 11.6
cDCs 18.6
Monocytes 37.8
DCs 58.1
NK cells 6.03
HLA-DR+ cells 26.2
Others 66.0
B cells 3.11
T cells 47.6
Others 46.2
CD8 7.90
CD4 84.5
CD123
CD11c
CD16
CD14
CD56
HLA-DR
CD19
CD3
CD8
CD4
B
Number of cells/mL of urine
D
Lymphocytes 40.6
Single Cells 98.5
Total Live+ cells 60.4
T cells 52.9
CD8 2.14
CD4 92.7
FSC-A
LD/CD19-BV510
CD3-AF700
CD8-BUV-395
CD4-APC-Cy7
C
Granulocytes
Monocytes
CD4+ T cells
CD8+ T cells
B cells
NK cells
mDCs
pDCs
% of cells
Naive
Experienced
E
CD4+ T cells
CD8+ T cells
unstimulated
BCG
PMA-Iono.
U03
U08
U11-2
U14
U18
TNFα-BV605
IFNγ-PE-Cy7
Urine-derived BCG-specific CD4+ T cells

Figure S2: Gating strategy and representative flow plots of urine immune cells and BCG-specific CD4+ T cells from patients with bladder cancer undergoing BCG therapy, related to Figure 3.

A. Representative flow cytometry gating strategy to identify the different immune cells present ex vivo in the urine of patients.

B. Number of cells per mL of urine across the different patients and urine specimens.

C. Immune cell composition in urine samples from BCG-therapy-naïve (n=16) and -experienced (n=6) NMIBC patients. Each dot represents one urine sample. Bars indicate medians. Statistical comparisons were performed using the Mann-Whitney test (two-tailed), as data were not normally distributed. *$p < 0.05$; **$p < 0.01$; ns, not significant.

D. Representative flow cytometry gating strategy to identify and characterize BCG-specific T cells in the urine of patients following polyclonal expansion and stimulation with BCG-peptide pool.

E. Intracellular cytokine staining to identify BCG-specific CD4+ T cells in urine samples. Frequency of TNFα and IFNγ (co-)expression are indicated.

Patient IDs are labeled as 'U' for urine samples, followed by the individual patient number.

Table S1: **Patient's demographics and clinical information**, related to Figure 1, 2 and 3.

Patient ID**	Age at diagnosis	Sex	Tumor stage	Treatment History
B01	71	M	pT1 high grade	Naive
B02	54	M	pTa high grade	Naive
B03	80	M	pT1 high grade	Naive
B04	74	M	pT1 high grade + CIS	Naive
B05	84	M	pT1 high grade	Naive
B06	59	M	pT1 high grade	Naive
B07	64	M	pTa high grade	Naive
B08	67	M	pT1 high grade	Naive
B09	74	M	pTa high grade	Naive
B10	62	M	pTa high grade	Naive
B11	59	M	pTa high grade	Naive
B12	74	M	pT1 high grade + CIS	Naive
B13	74	M	pTa high grade	Naive
B14	70	M	pTa high grade	Naive
U01	83	M	pT1 high grade	Naive
U02	83	F	CIS	BCG
U03	76	M	pT1 high grade	Naive
U04	77	M	pT1 high grade + CIS	Naive
U05	60	F	pTa high grade	Naive
U06	73	M	pTa low risk	BCG, MMC**
U07	86	M	pTa high grade + CIS	Naive
U08	81	M	pT1 high grade	Naive
U09	73	M	pTa high grade + CIS	Naive
U10	76	M	pTa high grade	Naive
U11	61	M	pT1 high grade	Naive
U12	83	M	pT1 high grade	BCG
U13	42	M	pTa high grade + CIS	Naive
U16	68	M	CIS	BCG
U15	77	M	pT1 high grade + CIS	Naive
U14	71	M	pTa high grade	BCG
U17	51	M	pTa high grade	Naive
U18	79	M	pTa high grade	Naive

**BXX = blood sample, UXX = urine sample, XX indicates the patient number ; **mitomycin C (MMC)*

Table S2: Patients, sample types and timepoints used per analysis, related to Figure 1, 2 and 3.

		Patients with detectable BCG-specific CD4+ T cells (**Figure 1C**)	BCG-specific CD4+ T cell frequency overtime (**Figure 1E**)	BCG-specific CD4+ T cell memory differentiation overtime (**Figure 1G,H**)	BCG-specific CD4+ T cell functionnal analysis (**Figure 2**)	Urine-derived pan-immune cell analysis (**Figure 3B-C**)	Urine-derived T cell expansion and detection of BCG-specific T cells (**Figure 3D-I**)
**Patient ID**	**Sample type**	**baseline ; induction**	**early ; late**	**early ; late**	**late**	**maintenance**	**maintenance**
B1	Frozen PBMC	W0 ; W4	W0 ; W4	W0 ; W4	W4		
B2	Frozen PBMC	W0 ; W2,W4,W5	W0 ; W4	W0 ; W4	W4		
B3	Frozen PBMC	W0 ; W3,W5	W0 ; W5	W0 ; W5	W5		
B4	Frozen PBMC	W0 ; W1,W3,W5	W1 ; W5	W1 ; W5	W5		
B5	Frozen PBMC	W0 ; W3,W4	W0 ; W4	W0 ; W4	W4		
B6	Frozen PBMC	*no baseline Tp ; W1,W3,W5*	W1 ; W3	W1 ; W3	W5		
B7	Frozen PBMC	*no baseline Tp ; W3*	*no early Tp ; W3*	*no early Tp ; W3*	W3		
B8	Frozen PBMC	W0 ; W1	W1 ; *no late Tp*	W1 ; *no late Tp*	*no late Tp*		
B9	Frozen PBMC	W0 ; W4	W0 ; W4	W0 ; W4	W4		
B10	Frozen PBMC	W0 ; W4	W0 ; W4	W0 ; W4	W4		
B11	Frozen PBMC	W0 ; W4	W0 ; W4	W0 ; W4	W4		
B12	Frozen PBMC	W0 ; W4	W0 ; W4	W0 ; W4	W4		
B13	Frozen PBMC	W0 ; W4	W0 ; W4	W0 ; W4	W4		
B14	Frozen PBMC	W0 ; W4	W0 ; W4	W0 ; W4	W4		
U01	Fresh Urine					W12	
U02	Fresh Urine					W11	
U03	Fresh Urine					W26	W26
U04	Fresh Urine					W27	
U05	Fresh Urine					W12	
U06	Fresh Urine					W26	
U07	Fresh Urine					W26	
U08	Fresh Urine					W13	W13
U09	Fresh Urine					W25	
U10	Fresh Urine					W11,W12,W13	
U11	Fresh Urine					W25,W27	W27
U12	Fresh Urine					W12	
U13	Fresh Urine					W27	
U14	Fresh Urine					W12	W12
U15	Fresh Urine					W13	
U16	Fresh Urine					W11,W12	
U17	Fresh Urine					W26	
U18	Fresh Urine					W13	W13
